# Polyphenon E Effects on Gene Expression in PC-3 Prostate Cancer Cells

**DOI:** 10.3390/ijms232214328

**Published:** 2022-11-18

**Authors:** L. Michael Carastro, Ethan J. Vallebuona, Ricardo Cordova, Ashely N. Gannon, Seung Joon Kim, Corrine M. Costello, Ricardo A. Declet-Bauzo, Nagi Kumar, Jong Y. Park

**Affiliations:** 1 Department of Chemistry, Biochemistry & Physics, College of Natural & Health Sciences, The University of Tampa, Tampa, FL 33606, USA; 2 Department of Internal Medicine, College of Medicine, The Catholic University of Korea, Seoul 06591, Republic of Korea; 3 Department of Cancer Epidemiology, Moffitt Cancer Center, Tampa, FL 33612, USA

**Keywords:** prostate cancer, polyphenon E, DNA microarray, gene expression, *MXD1*, *RGS4*

## Abstract

Polyphenon E (Poly E) is a standardized, caffeine-free green tea extract with defined polyphenol content. Poly E is reported to confer chemoprotective activity against prostate cancer (PCa) progression in the TRAMP model of human PCa, and has shown limited activity against human PCa in human trials. The molecular mechanisms of the observed Poly E chemopreventive activity against PCa are not fully understood. We hypothesized that Poly E treatment of PCa cells induces gene expression changes, which could underpin the molecular mechanisms of the limited Poly E chemoprevention activity against PCa. PC-3 cells were cultured in complete growth media supplemented with varied Poly E concentrations for 24 h, then RNA was isolated for comparative DNA microarray (0 vs. 200 mg/L Poly E) and subsequent TaqMan qRT-PCR analyses. Microarray data for 54,613 genes were filtered for >2-fold expression level changes, with 8319 genes increased and 6176 genes decreased. Eight genes involved in key signaling or regulatory pathways were selected for qRT-PCR. Two genes increased expression significantly, *MXD1* (13.98-fold; *p* = 0.0003) and *RGS4* (21.98-fold; *p* = 0.0011), by qRT-PCR. *MXD1* and *RGS4* significantly increased gene expression in Poly E-treated PC-3 cells, and the *MXD1* gene expression increases were Poly E dose-dependent.

## 1. Introduction

Prostate cancer (PCa) is the most prevalent non-cutaneous cancer diagnosis among men in the United States [1]. According to SEER data, there will be an estimated 268,490 new PCa cases diagnosed and 34,500 deaths attributed to PCa in 2022 [2]. Although the mortality rate for PCa is relatively low compared to some other cancers, e.g., lung and pancreas, the relatively high frequency of occurrence, challenges in screening and early diagnosis, as well as variability in the efficacy of treatment modalities raises the value of potential PCa chemoprevention strategies. Polyphenon E (Poly E) is a proprietary, caffeine-free standardized blend of polyphenolic compounds found in green tea, and consists of ~90% catechins, primary epigallocatechin-3-gallate (EGCG), which comprises >65% EGCG, <10% (-)-epigallocatechin, <10% (-)-epicatechin, <10% (-)-epicatechin gallate, and other trace amounts of catechin derivatives. It was approved by the US FDA as an effective treatment against external genital and perianal warts [3,4,5]. In addition, the anti-cancer and anti-inflammation properties of green tea polyphenols found in Poly E have previously been reviewed [6,7]. Green tea polyphenols (GTPs) in Poly E confer chemopreventive benefits against PCa, in part by affecting the gut microbiome [8]. Polyphenolic compounds found in green tea and Poly E have antioxidant effects and modulate cellular miRNAs that both aid in cancer prevention [9]. We previously reported significant, dose-dependent reductions in prostate tumor burdens, as well as increased life spans, in TRAMP mice exposed to Poly E in drinking water [10]. A human clinical trial using Poly E oral administration found a significant chemopreventive benefit against developing PCa, but only in those previously diagnosed with atypical small acinar proliferation (ASAP) or high-grade prostate intraepithelial neoplasia (HGPIN) [11]. Therefore, there is evidence to suggest that Poly E is a potentially effective intervention strategy to reduce the risk of PCa, at least in cases where the patient has already been diagnosed with ASAP or HGPIN.

The major polyphenolic constituent of Poly E, EGCG, has been reported to regulate several key cellular signaling pathways [12,13,14], proinflammatory factors [15,16], and apoptosis [17,18]. It has been suggested that EGCG is less effective alone in its beneficial health properties than it is in conjunction with the other polyphenolic compounds naturally found in green tea, and in Poly E [19]. Although numerous reports have studied the molecular mechanisms underpinning the cancer-preventive activities of EGCG, few have explored the mechanisms specific to Poly E [3]. Thus, the chemopreventive mechanisms of GTPs, but more so for Poly E, are not fully understood.

We hypothesized that Poly E treatment of prostate cancer cells in culture induces gene expression changes, which could provide insights into the molecular mechanism(s) of potential Poly E chemopreventive activity against PCa. The purpose of this study was to identify genes that experienced substantially altered gene expression levels, with changes >2-fold in PC-3 PCa cells after Poly E treatment, and to validate these findings using qRT-PCR methodologies.

## 2. Results

### 2.1. Poly E Treatment of PC-3 Cell Cultures

**Figure 1:**
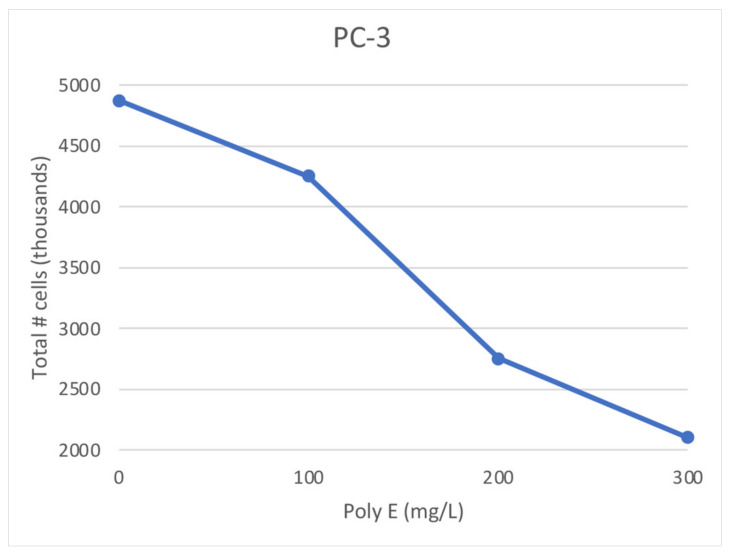
Polyphenon E-treated PC-3 cell growth curve. PC-3 cell cultures were initiated with equal numbers of cells, and were cultured in complete growth media at the indicated concentrations of Poly E for 24 h. After 24 h of Poly E treatment, PC-3 cells were collected by centrifugation, resuspended in 1× PBS, and total cell numbers in each Poly E treatment were determined by measuring cell concentrations using an automated TC-20 cell counter (Bio-Rad, Hercules, CA, USA).

PC-3 prostate cancer cells were cultured in complete growth media supplemented with Poly E at varied concentrations for 24 h. Cultured cells were then collected and washed with 1× PBS, and cell concentrations were determined. There was a consistent pattern of decreasing cell growth with increasing Poly E concentrations (Figure 1). The 200 mg/L Poly E concentration treatment corresponded, approximately, to a 50% reduction in cell populations compared to untreated (0 mg/L Poly E) for the PC-3 (Figure 1; 0 mg/L vs. 200 mg/L Poly E) cell line.

### 2.2. DNA Microarray Analyses of Poly E-Treated PC-3 Cells

**Figure 2:**
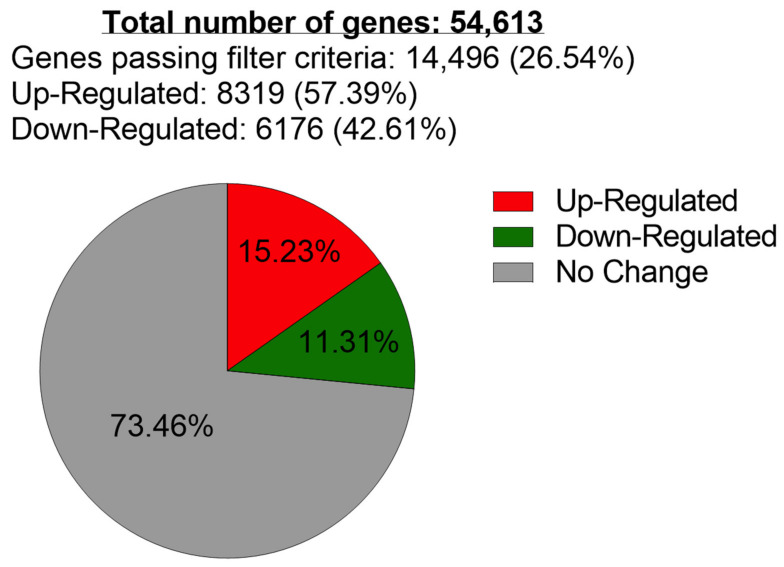
Poly E-treated PC-3 cell microarray gene expression filtering analysis. Pie chart presentations of gene filtering analysis, with genes <2-fold expression-level change (in GREY), genes > 2-fold expression-level increase (in RED), and >2-fold expression level decrease (in GREEN).

The Medium-level Poly E (200 mg/L) concentration tested had total cell numbers that corresponded to ~50% of the number of cells in the Poly E-untreated (0 mg/L) samples (Figure 1); therefore, these two samples were used for comparative DNA microarray analysis. RNA samples isolated from Poly E-untreated (0 mg/L) PC-3 cells, as well as from the Medium-Poly E- (200 mg/L) treated PC-3 cell samples, were delivered to the Moffitt Cancer Center Genomic Core Facility (Tampa, FL, USA) for microarray analysis. The resulting .CEL data files were analyzed using Applied Biosystems’s Transcriptome Analysis Console 4.0, using a >2-fold gene expression change filter to generate Figure 2. Of the 54,613 genes in PC-3 cells analyzed by DNA microarray, we found 8319 genes with increased expression levels >2-fold and 6176 genes with decreased expression >2-fold (Figure 2).

**Figure 3:**
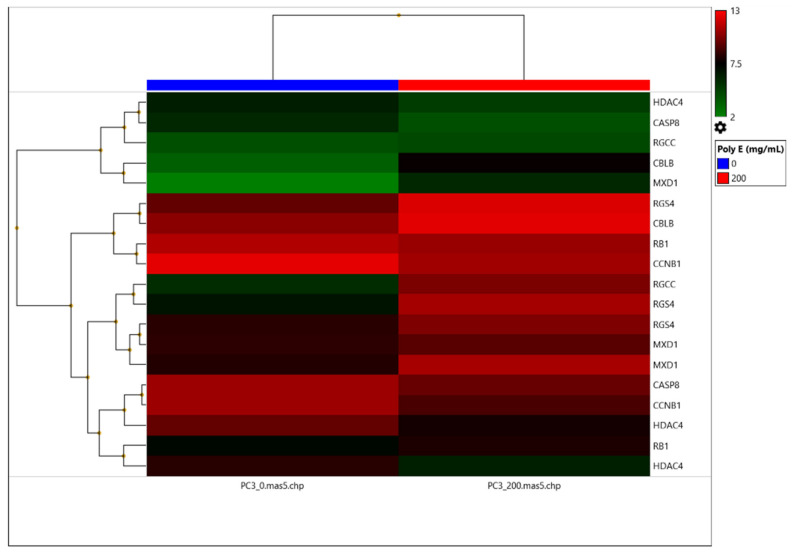
Poly E-treated PC-3 cell line microarray gene expression hierarchical clustering. Reporter probe signal intensities of the eight genes selected for qRT-PCR analysis are labeled with red representing upregulation and green representing downregulation in gene expression, relative to the middle-adjusted intensity depicted in black. A scale of signal intensity values ranging from 2 (lowest) to 13 (highest) is depicted at the top right of the heat map. Columns represent either untreated (**left**; 0 mg/L) or Poly E-treated (**right**; Mid 200 mg/L). Rows represent individual Affymetrix probes with gene symbols displayed to the right of each row. Hierarchical clustering of the genes analyzed is indicated by the nodal connections (left side of figure). Gene symbols (right side of figure) with their corresponding Affymetrix probe set IDs (from top to bottom of list): *HDAC4* (1554322_a_at), *CASP8* (1553306_at), *RGCC* (239827_at), *CBLB* (208348_s_at), *MXD1* (206877_at), *RGS4* (204337_at), *CBLB* (209682_at), *RB1* (203132_at), *CCNB1* (214710_s_at), *RGCC* (218723_s_at), *RGS4* (204338_s_at), *RGS4* (204339_s_at), *MXD1* (226275_at), *MXD1* (228846_at), *CASP8* (213373_s_at), *CCNB1* (228729_at), *HDAC4* (204225_at), *RB1* (211540_s_at), and *HDAC4* (228813_at).

Seven genes (*CASP8, CBLB, CCNB1, HDAC4, MXD1, RGCC,* and *RGS4*) involved in key signaling pathways were selected from the DNA microarray data after filtering for >2-fold gene expression change filter, and are listed with the corresponding values of the relative fold-change in gene expression for each Affymetrix probe (Table 1). Although it did not pass the DNA microarray > 2-fold gene expression change filtering analysis, the *RB1* gene was included in the subsequent analyses to serve as a control. Because *RB1* had <2-fold changes in gene expression by microarray (Table 1), it would not be expected to have a > 2-fold change in gene expression by qRT-PCR analysis, at least at the Medium Poly E treatment level used for the microarray. Therefore, a total of eight genes (*CASP8*, *CBLB*, *CCNB1*, *HDAC4*, *MXD1*, *RB1*, *RGCC*, and *RGS4*) were included in the heat map analysis of the microarray data (Figure 3), with numeric values for the gene expression changes from microarray data, using various types of probe sets for each gene, also presented in a tabular format (Table 1).

### 2.3. Quantitative Real Time-PCR

**Figure 4:**
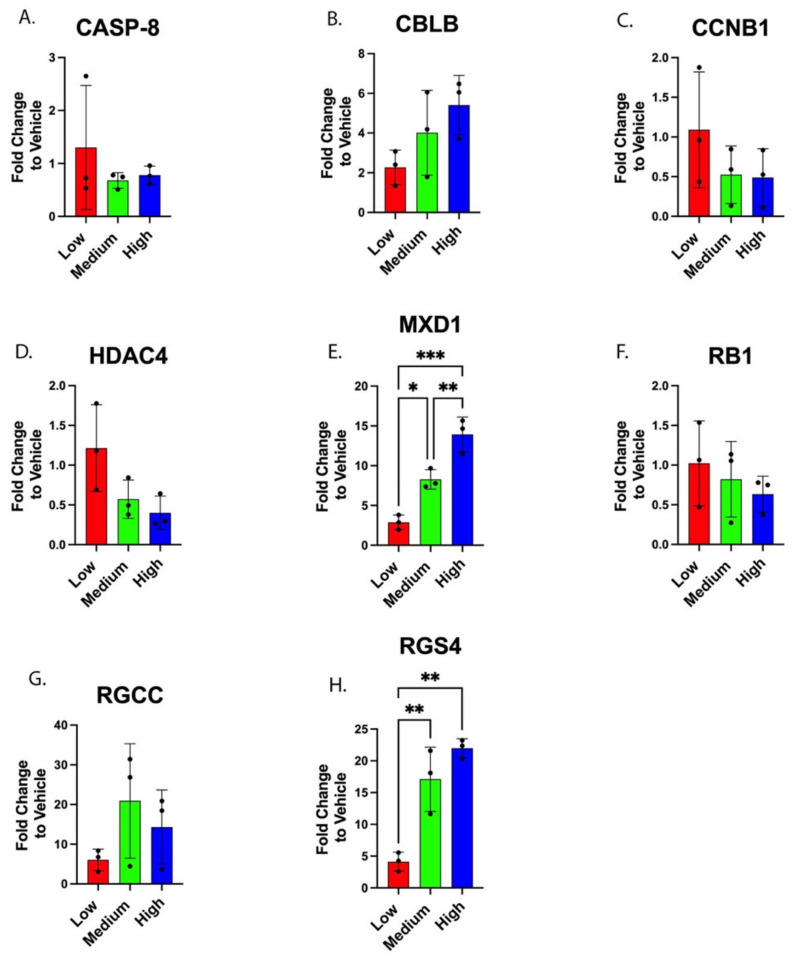
TaqMan qRT-PCR analyses of gene expression in Poly E-treated PC-3 cells. Histogram plots of the gene expression fold changes for the eight genes (**A**–**H**) analyzed by TaqMan qRT-PCR compared to “vehicle” Poly E untreated (0 mg/L) samples. Each graphic has the specific gene targeted indicated above the histogram. Statistically significant gene expression changes between treatment groups are indicated by asterisks. For *MXD1* in Figure 4E: * *p*-value = 0.012, ** *p*-value = 0.001, *** *p*-value = 0.0003. For *RGS4* in Figure 4H: low vs. medium ** *p*-value = 0.006, low vs. high ** *p*-value = 0.001. Poly E concentrations are indicated by low = 100 mg/L, medium = 200 mg/L, and high = 300 mg/L.

Eight genes (*CASP8*, *CBLB*, *CCNB1*, *HDAC4*, *MXD1*, *RB1*, *RGCC*, and *RGS4*) were selected from DNA microarray data for more comprehensive qRT-PCR gene expression analyses of PC-3 cells treated with varied Poly E-treated concentrations (Figure 4). Cellular RNA samples were converted into cDNAs for templates in qRT-PCR analyses using the ΔΔCt method, with ACTB as the endogenous control. The qRT-PCR results for six of the eight genes analyzed did not discern significant differences (*p*-values > 0.05) in gene expression changes between the Poly E treatment groups (low, medium, high), including *CASP8* (Figure 4A), *CBLB* (Figure 4B), *CCNB1* (Figure 4C), *HDAC4* (Figure 4D), *RB1* (Figure 4F), and *RGCC* (Figure 4G). However, the observed decrease in *HDAC4* gene expression between low- to high-Poly E concentrations (Figure 4D) was a 0.33-fold change, which approached significance (*p*-value = 0.076). The mean value decrease in *HDAC4* gene expression at the High-Poly E concentration, compared to untreated (vehicle only), was 0.40-fold (Figure 4D, High Poly E). The two Poly E dose-dependent increases observed in *CBLB* gene expression (Figure 4B, low vs. medium and medium vs. high) were not significant (*p*-values = 0.158 and 0.838, respectively), even though the increase in the mean values of gene expression fold-change between the low- and high-Poly E concentrations (Figure 4B, Low vs. High) was 2.38-fold (*p*-value = 0.113). At the highest Poly E concentration tested, the *CBLB* mean value for gene expression change was 5.41-fold compared to Poly E untreated PC-3 cells (Figure 4B, High). The fold changes in *RGCC* gene expression (Figure 4G, low vs. medium and medium vs. high) were not significant (*p*-values = 0.244 & 0.715, respectively) between Poly E treatment groups; however, the highest mean value increase in *RGCC* gene expression was 20.91-fold at the medium Poly E concentration. Three genes (*CASP8*, *CCNB1*, and *RB1*) lacked significance in the changes in gene expression levels between Poly E treatment groups, and had mean values of gene expression fold-changes of <2-fold.

Two genes did experience significant expression changes in response to Poly E treatment: *MXD1* (Figure 4E) and *RGS4* (Figure 4H). *MXD1* gene expression increases induced by Poly E treatment were dose-dependent (Figure 4E, low vs. medium and medium vs. high) and significant (*p*-values = 0.012 and 0.001, respectively). The increase in the gene expression fold-change between the low and high Poly E concentrations (Figure 4E, Low vs. High) was 4.86-fold, and was highly significant (*p*-value = 0.0003). At the highest Poly E treatment, the mean value of *MXD1* gene expression increased by 13.93-fold (Figure 4E, High). *RGS4* gene expression increased up to a mean value of 17.11-fold (Figure 4H, High) in PC-3 cells treated at the highest Poly E level. The 4.15-fold increase in *RGS4* gene expression mean values between the low vs. medium Poly E treatment levels (Figure 4H, low vs. medium) was significant (*p*-value = 0.006), and the *RSG4* gene expression, which increased by 5.33 between the low and high Poly E concentrations (Figure 4H, low vs. high), was significant (*p*-value = 0.001).

## 3. Discussion

### 3.1. Comparison of Microarray and Quantitative Real Time-PCR Data

The qRT-PCR gene expression fold-changes from medium-Poly E treatments (Figure 4A–H, Medium) are largely consistent with the fold-change gene expression values from the DNA microarray data (Table 1). There are inherent differences in these two types of gene expression analyses, DNA microarray and qRT-PCR. In the DNA microarray analyses of gene expression changes, there are different types of probe sets being used to hybridize with target gene sequences. The probes with the “_at” suffix are specific to a slicing isoform of a single gene, whereas those ending in “a_at” or “s_at” are less specific. The “a_at” probe sets hybridize to multiple splicing isoforms from a single gene, but the “s_at” probe sets will hybridize to a target sequence found in multiple genes and belonging to a related family of genes. However, for all the qRT-PCR data collected in this study, the “Best Coverage” TaqMan primer–probe sets were utilized, which are expected to detect the widest variety of gene isoforms. Four genes (*CASP8*, *CCNB1*, *HDAC4*, and *RB1*) had gene expression level changes detected by multiple microarray probe sets that ranged from just under 2-fold to no more than 3.5-fold (Table 1), whereas the qRT-PCR mean values of fold-changes in gene expression at the medium Poly E treatment level were 0.68-fold for *CASP8* (Figure 4A, medium), 0.52-fold for *CCNB1* (Figure 4C, medium), 0.57-fold for *HDAC4* (Figure 4D, medium), and 0.82-fold for *RB1* (Figure 4F, medium). All of the fold change values observed by microarray (Table 1) were within the range of gene expression fold-change values used to determine mean values of the medium Poly E treatments (Figure 4A,C,D,F at Medium Poly E). In the *CASP8* microarray fold-change data (Table 1) the data for the two probes used were near in value, but the 1553306_at probe set had a decreased gene expression fold change at −3.2, which is a slightly larger change than the −2.23-fold change reported that is less specific. *CCNB1* and *HDAC4* had a similar result in the microarray analysis, in that the different types of probe sets reported similar fold changes in gene expression (Table 1, *CCNB1* vs. *HDAC4*). The *RB1* probe sets used had fold changes < 2-fold, but one indicated 1.85-fold increased gene expression (Table 1, *RB1* probe set 211540_s_at), while the other detected a −1.43-fold decrease in gene expression (Table 1, *RB1* probe set 203132_at). As the latter *RB1* probe set is more specific, the data from this probe were compared to the qRT-PCR gene expression data that indicated a 0.82-fold decreased expression (Figure 4, medium).

Likewise, four genes that did experience > 2-fold gene expression changes by microarray (Table 1; *CBLB*, *MXD1*, *RGCC*, and *RGS4*) were also in general agreement with the microarray data for these genes at the medium Poly E treatment level (Figure 4B,E,G,H; Medium Poly E). *CBLB* microarray analysis registered a 19.01-fold gene expression change (Table 1, *CBLB* probe set 208348_s_at) with the less specific probe set, but a 3.83-fold increase in gene expression with the more specific probe set (Table 1, *CBLB* probe set 209682_at), which is quite close to the qRT-PCR-detected mean value showing a 4.01-fold increase in *CBLB* gene expression. This suggests that the *CBLB* mRNA isoform(s) are detected by the Probe #209682_at. *MXD1* microarray data indicated the use of three different isoform-specific probe sets (Table 1, *MXD1*), but the first, listed with a 13.36-fold increase in gene expression, is very close to the 13.93-fold increase mean value of *MXD1* gene expression change observed by qRT-PCR (Figure 4E, Medium). *RGCC* gene expression changes detected by microarray analysis suggest that the less specific “gene family” probe set detected an increased expression of 23.12-fold (Table 1, *RGCC* probe set 218723_s_at), while the *RGCC* isoform-specific probe detected only a 1.22-fold increase in gene expression (Table 1, *RGCC* probe set 239827_at). The former microarray probe set detected a 23.12-fold increase in *RGCC* gene expression, which is in better agreement with the qRT-PCR 20.91-fold mean value increase in *RGCC* gene expression (Figure 4G, medium). Thus, in the case of *RGCC*, it seems that the *RGCC* isoform with increased expression detected in PC-3 cells by DNA microarray and qRT-PCR was not detected by the more specific microarray *RGCC* probe set. Lastly, *RGS4* microarray data reported gene expression changes for three probe sets: two were less specific gene family probes (Table 1, *RGS4* probes 204338_s_at and 204339_s_at) with different levels of increase in fold-changes for *RGS4* expression at 20.54-fold and 3.69-fold, respectively. The only *RGS4* isoform-specific probe indicated a 6.12-fold increase in *RGS4* expression (Table 1, probe set 204337_at). Given that qRT-PCR detected a 17.11-fold increase in *RGS4* gene expression (Figure 4H, medium), the qRT-PCR increase in *RGS4* gene expression could correlate with some combination of hybridizations from the two *RGS4* microarray probes that detected 20.54-fold and 6.12-fold increases in *RGS4* expression.

### 3.2. Implications for Poly E-Induced Gene Expression Changes in PC-3 Cells

Poly E is composed of polyphenols found in green tea, but it primarily (>65%) consists of EGCG. EGCG and other GTPs have been reported to alter expression of several genes. For example, using DURPO and LNCaP PCa cells as a model system, it was demonstrated that green tea polyphenol mixture (GTP) and EGCG treatments at 10 mg/L and 20 mM, respectively, induced the epigenetic reactivation and expression of tissue inhibitors of matrix metalloprotease-3 (*TIMP-3*), while decreasing the expression of enhancers of zeste homoloug 2 (*EZH2*) and its catalytic product, trimethylated histone H3, at lysine 27 (H3K27me3) [20]. However, the data reported herein found that *HDAC4* gene expression did not change by more than 3-fold as measured by qRT-PCR (Figure 4G), but the microarray data did predict that *HDAC4* differential gene expression changes for primary prostate cells would *HDAC4* increase by nearly 7-fold versus DU145 and PC-3 cells, which experienced 2-to-5-fold decreases in *HDAC4* expression (Table 1).

Of the eight genes selected after microarray and analyzed by qRT-PCR in this study, only *MXD1* (Figure 4E) and *RGS4* (Figure 4H) had gene expression changes that were significantly affected by Poly E treatment in PC-3 cells. *MXD1* is a tumor suppressor gene (TSG), and its primary cellular role is to inhibit G1-phase cell cycle progression through negative regulation of the myc-E2F axis [21,22]. Low *MXD1* mRNA expression levels were reported in patients with esophageal squamous cell carcinoma [23]. Treatment of three PCa cell lines, including PC-3 cells, with cardiac glycoside deslanoside resulted in an upregulation of *MXD1* that positively correlated to overall PCa patient survival [24]. The dose-dependent increase in *MXD1* expression observed here in response to Poly E treatment reached a mean value of 13.93-fold by qRT-PCR (Figure 4E, High). It could, conceivably, inhibit cell cycle progression and PCa cell growth in vivo, as it has in our PCa cell line-based study. Additionally, *RGS4* is reported to be a negative regulator of G protein-coupled receptor (GPCR) signaling [25,26]. In human non-small cell lung cancer (NSCLC), *RGS4* has been identified as a novel tumor suppressor with prognostic potential [27]. *RGS4* was identified as having a cell growth suppression function in glioblastoma [28], thyroid [29], and ovarian cancers [30]. Additionally, *RGS4* has been reported as a potential therapeutic target for suppressing proliferation and invasion of A375 melanoma cells [31]. Microarray data evaluating the transcriptomes of patient derived cancer tissue samples suggested a role of RGS family proteins in the progression of various types of cancers [32]. Currently, no research exists linking *RGS4* to human PCa, but *RGS2* has been reported to be significantly downregulated in LNCaP cells and CWR22Rv1 PCa cellular models [33]. The increase, observed herein, in the expression of *RGS4* in PC-3 cells after Poly E treatment reached a mean value of 17.11-fold (Figure 4H, high) and could suggest a role for *RGS4* in attenuating GPCR-mediated cell proliferation in PC-3, and perhaps other in PCa cells as well. These two findings we report here for *MXD1* and *RGS4* provide potential molecular mechanisms that could explain observed decreases in PC-3 cell proliferation in response to Poly E exposure. Furthermore, these two potential molecular mechanisms are not mutually exclusive, and have the capacity to be additive, or even synergistic, in their antiproliferative effects.

### 3.3. Study Limitations and Future Experimental Directions

One limitation of this study is the use of the PC-3 cells as the model system. Future experiments paralleling those reported herein will be performed using other established PCa cell lines, e.g., DU145 and LNCaP, as well as non-cancerous primary prostate cells. Using three different PCa cell lines and primary prostate cells could be informative in detecting differences in Poly-E induced gene expression changes between PCa cell lines and normal cells. Other experimental approaches which we plan to utilize in these future experiments include performing hierarchical clustering analyses to aid in identifying gene for subsequent qRT-PCR analyses. In addition, we plan to perform rescue experiments by depleting mRNAs of target genes experiencing Poly E-induced increases in gene expression, e.g., *MXD1* and *RGS4*, using siRNA approaches in cell culture models (PC-3, DU145, LNCaP, primary prostate cells). Furthermore, additional analyses in future works can include cell cycle analyses using propidium iodide staining, coupled with FACS analyses methodologies and Annexin V staining, to assess apoptosis status. Lastly, we plan to conduct protein expression studies using Western blotting approaches to assess whether the gene expression changes observed at the level of transcripts can also be observed at the protein level.

Collectively, these data presented could provide insights into, or new avenues for, the development of biomarkers or therapeutic interventions for PCa. Presumably, prostate tumor cells that have lost the ability to express functional *MXD1* or *RGS4* would lose the capacity to enforce these observed Poly E-mediated antiproliferative effects on PC-3 cells, and perhaps other PCa cells as well. If true, this could provide the basis for novel screening tools to identify those patients with prostate tumor cells who might respond to Poly E treatment.

## 4. Materials and Methods

### 4.1. PC-3 Cell Cultures and Poly E Treatments

PC-3 human prostate cancer cell line was purchased (ATCC (Manassas, VA, USA), CRL-1435™), and cells were cultured in F12 Ham’s nutrient mixture media (Hyclone (Logan, UT, USA), cat#SH30026.01) supplemented with 10% fetal bovine serum (Hyclone, cat#SH30071.03), 1× Anti-Anti (Gibco; ref#15240-062), and 10 µg/mL Ciproflaxin (bioPLUS Chemicals (Kuala Lumpur, Malaysia), sku#40310031-1) at 37 °C under 5% CO_2_. Poly E was solubilized in sterile water, at 1000× the Poly E-treatment concentration, prior to supplementing complete F12 culture media. Upon reaching 80% confluence, PC-3 cell culture media was changed to complete F12 media supplemented with Poly E at one of the Poly E concentrations tested (untreated = 0 mg/L, low = 100 mg/L, medium = 200 mg/L, high 300 mg/L) for 24 h. The Poly E concentrations and 24 h treatment time used were based on a published report using Poly E to treat PC-3 cells [34]. Untreated (0 mg/L) cell culture media was supplemented with vehicle (water) only. After 24 h of Poly E treatments, cells were washed with 1× PBS (without Ca, Mg), liberated from the culture dishes with Trypsin-EDTA for 5 min, inactivated with complete F12 media, and collected by centrifugation at 200× *g* (4 C) for 5 min. Media was removed from cell pellets and cells were resuspended in 1× PBS (without Ca, Mg). Cell numbers were determined using a TC-20 (Bio-Rad, Hercules, CA, USA) automated cell counter, and cells were centrifuged as before. PBS was removed and PBS-washed cell pellets were frozen at −80 °C until RNA isolation.

### 4.2. DNA Microarray

RNA samples were isolated from PC-3 cells using RNeasy PLUS Mini Prep kits (Qiagen, Hilden, Germany) according to the manufacturer’s protocol, and were assessed for quantity and quality using a spectrophotometer. RNA samples were delivered to the Molecular Genomics Core Facility at Moffitt Cancer Center (Tampa, FL, USA) for microarray analyses. RNA samples were subjected to QC screening with a Bioanalyzer RNA chip using TapeStation Analysis Software A.02.01 (Agilent Technologies, Santa Clara, CA, USA). After passing QC analyses, RNAs were processed and hybridized to Affymetrix Human U133 Plus 2.0 array, and .CEL files were generated. Microarray .CEL data files were aquired from the Molecular Genomics Core Facility at Moffitt Cancer Center (Tampa, FL, USA). The .CEL data contained unadjusted probe intesities corresponding to the probeset contained in the GeneChip^TM^ Human Genome U133 Plus 2.0 Array platform, and loaded into Applied Biosystems’s (Thermo Fisher Scientific, Waltham, MA, USA) Transcriptome Analysis Console 4.0.2.15 (TAC 4.0). Expression (Gene) analysis of .CEL data files for PC-3 cells was performed individually, with the comparison factors being 0 and 200 mg/L of Polyphenon E exposure after 24 h. Following annotation of the experimental factors within the console, the expression analysis was run utilizing .MAS5 summarization to normalize each array sequentially, with an additonal log base 2 adjustment step of the yielded intensity values. The expression data were reported in a gene table filtered by the criteria of <−2 or >2-fold change values in gene expression, with the data displayed in hierarchical clustering (heat map) and parts of a whole analysis (pie chart) of target Affymetrix probe sets for specified genes of interest (TAC 4.0’s “Gene View” tab). The criteria for choosing a target gene of interest included known involvement in oncogenic mechanisms associated with PCa or notably high differences in signal intensities between the untreated (control, 0 mg/L) and treated (medium, 200 mg/L) comparison groups.

### 4.3. Quantitative Real-Time PCR

RNA samples were isolated from PC-3 cells and analyzed as before (in DNA microarray). RNA samples (1 microgram) were converted into cDNAs using SuperScript^TM^ VILO^TM^ cDNA Synthesis Kit (Invitrogen, Waltham, MA, USA). cDNA samples were used as templates in TaqMan qRT-PCR reactions performed with a StepOne Real-Time PCR Plus system (Applied Biosystems™, Waltham, MA, USA). Each qRT-PCR reaction (20 microL) was performed in triplicate using 10 ng of cDNA, 1× TaqMan™ Gene Expression Master Mix (Life Technologies, Carlsbad, CA, USA), and 1× TaqMan^®^ real time PCR “Best Coverage” gene expression probes (Applied Biosystems™) were utilized in each qRT-PCR reaction for the following gene targets: *RGS4* (Hs01111690_g1), *CASP8* (Hs06630780_s1), *CBLB* (Hs00180288_m1), *CCNB1* (Hs01030099_m1), *RB1* (Hs01078066_m1), *HDAC4* (Hs01041648_m1), *RGCC* (Hs00204129_m1), and *MXD1* (Hs00965581_m1). The StepOnePlus™ qRT-PCR system was programmed according to the following cycling parameters: 2 min of initial denaturation at 50 °C, followed by a single 10 min period at 95 °C to facilitate Uracil-D-Glycosylase activity before 40 cycles of 15 s at 95 °C, then 1 min at 60 °C. Relative quantification using the comparative CT method was used to calculate ΔΔCt values normalized to mRNA expression levels of an endogenous control gene: *ACTB* (Hs01060665_g1). Three independent biological experiments were conducted in order to collect qRT-PCR data sets, which were performed using technical triplicates for each qRT-PCR reaction.

### 4.4. Statistical Analysis

Statistical comparisons of mean ± standard deviation was performed using the Student *t* test in GraphPad Prism 9.4.1 (GraphPad Software, San Diego, CA, USA), and statistical significance between multiple groups was determined using one-factor ANOVA followed by Tukey’s HSD test. Statistical significance was established if the computed *p*-value was less than 0.05 (α).

## 5. Conclusions

*MXD1* (13.98-fold; *p* = 0.0003) and *RGS4* (21.98-fold; *p* = 0.0011) significantly increased expression in Poly E-treated PC-3 cells, and the *MXD1* gene expression increases were Poly E dose-dependent.

## Figures and Tables

**Table 1:** DNA microarray data with changes in gene expression for Poly E-treated PC-3 cells. Affymetrix GeneChipTM Human Genome U133 Plus 2.0 Array platform probe sets targeting the 8 identified genes of interest. A gene table of log fold change values, depicting individual Column 1, represents Affymetrix Probe IDs, where probe sets denoted with the suffix “_s_at” represent probes in an identical probe set, which hybridize multiple transcripts for separate genes. Probe sets denoted with the suffix “_a_at” hybridize with multiple transcripts arisen from a single gene, and probe sets denoted with the suffix “_x_at” correspond to a mixed probe set which has potential of cross hybridization with sequences other than the target. Probe sets with only the suffix “_at” contain identical probes that perfectly hybridize with only a single unique sequence in a single transcript. Column 3 displays log2 fold-change values, describing how much the measured signal changes for a probe set from the untreated (control; 0 mg/L Poly E) to the medium treatment (mid; 200 mg/L Poly E) group.

Affymetrix Probe ID	Gene Symbol	Log_2_-Fold Change
213373_s_at	*CASP8*	−2.23
1553306_at		−3.2
208348_s_at	*CBLB*	19.01
209682_at		3.83
214710_s_at	*CCNB1*	−2.77
228729_at		−3.47
1554322_a_at	*HDAC4*	−2.47
204225_at		−3.23
228813_at		−4.45
206877_at	*MXD1*	13.36
228846_at		7.35
226275_at		1.91 *
211540_s_at	*RB1*	1.85 *
203132_at		−1.43 *
218723_s_at	*RGCC*	23.12
239827_at		1.22 *
204338_s_at	*RGS4*	20.54
204337_at		6.12
204339_s_at		3.69

* Log_2_-fold gene expression increase/decrease <2-fold.

## Data Availability

The data presented in this study are available upon request from the corresponding author.
